# Deep Genome Resequencing Reveals Artificial and Natural Selection for Visual Deterioration, Plateau Adaptability and High Prolificacy in Chinese Domestic Sheep

**DOI:** 10.3389/fgene.2019.00300

**Published:** 2019-04-02

**Authors:** Weimin Wang, Xiaoxue Zhang, Xiang Zhou, Yangzi Zhang, Yongfu La, Yu Zhang, Chong Li, Youzhang Zhao, Fadi Li, Bang Liu, Zhihua Jiang

**Affiliations:** ^1^College of Animal Science and Technology, Gansu Agricultural University, Lanzhou, China; ^2^Key Laboratory of Agricultural Animal Genetics, Breeding and Reproduction of Ministry of Education, Huazhong Agricultural University, Wuhan, China; ^3^Department of Animal Sciences, Washington State University, Pullman, WA, United States; ^4^The State Key Laboratory of Grassland Agro-Ecosystems, College of Pastoral Agriculture Science and Technology, Lanzhou University, Lanzhou, China; ^5^Engineering Laboratory of Sheep Breeding and Reproduction Biotechnology in Gansu Province, Minqin, China

**Keywords:** sheep, domestication, whole-genome resequencing, vision, adaptability

## Abstract

Sheep were one of the earliest domesticated animals. Both artificial and natural selection during domestication has resulted in remarkable changes in behavioral, physiological, and morphological phenotypes; however, the genetic mechanisms underpinning these changes remain unclear, particularly for indigenous Chinese sheep. In the present study, we performed pooled whole-genome resequencing of 338 sheep from five breeds representative of indigenous Chinese breeds and compared them to the wild ancestors of domestic sheep (Asian mouflon, *Ovis orientalis*) for detection of genome-wide selective sweeps. Comparative genomic analysis between domestic sheep and Asian mouflon showed that selected regions were enriched for genes involved in bone morphogenesis, growth regulation, and embryonic and neural development in domestic sheep. Moreover, we identified several vision-associated genes with funtional mutations, such as *PDE6B* (c.G2994C/p.A982P and c.C2284A/p.L762M mutations), *PANK2*, and *FOXC1/GMSD* in all five Chinese native breeds. Breed-specific selected regions were determined including genes such as *CYP17* for hypoxia adaptability in Tibetan sheep and *DNAJB5* for heat tolerance in Duolang sheep. Our findings provide insights into the genetic mechanisms underlying important phenotypic changes that have occurred during sheep domestication and subsequent selection.

## Introduction

The domestication of wild animals to meet humans needs as well as changes in the natural environment is an important aspect of the history of modern human civilization. Sheep are one of most economically important species of farmed animals in the world, providing a variety of products that are important to human society, such as meat, milk, and wool. Sheep were one of the earliest animals to be domesticated during early Neolithic period after dogs and goats (Zeder, 2008). After domestication in the Fertile Crescent approximately 8,000–11,000 years ago, sheep spread to every corner of the globe alongside their human domesticators (Legge, 1996; Vigne et al., 2005). Despite genetic studies, the identity of the wild ancestor of the domestic sheep (*Ovis aries*) remains to be determined; however, several species or subspecies of the genus *Ovis* have been suggested, including urial (*Ovis vignei*), argali (*Ovis ammom*) and Asian mouflon (*Ovis orientalis*), which are considered to be the most probable candidates (Rezaei et al., 2010; Sanna et al., 2015). Both *Ovis aries* and *Ovis orientalis* are interfertile; and compared with the other wild Asian sheep species, only *Ovis orientalis* has the same chromosome complement (2n = 54) as that of the domestic sheep (*Ovis aries*) (Nalder et al., 1973).

The evolution of the domestic sheep has been associated with marked phenotypic changes in behavior, reproduction, and growth. Identifying the genetic changes underlying these phenotypic changes should help to clarify the genetic mechanism of successful domestication of animals as well as practical breeding strategies for the improvement of sheep productivity and resilience to environmental challenges. Compared with studies on the domestication of dogs (Axelsson et al., 2013), chickens (Wang et al., 2016), pigs (Wang et al., 2015; Li et al., 2017), and cattle (Kim et al., 2017), relatively little is known about the changes associated with sheep domestication. A pioneer study by Yang et al. (2016) used resequencing to obtain 77 sheep genomes from different ecological environments and identify several selective sweeps associated with adaptations to extreme environments. Subsequently, Zhao et al. (2017) described artificial selection for tail type, coat color, and wool quality based on SNP BeadChip data. Despite the rapid development of genetic technology and identification of several selective sweeps associated with important phenotypes, such as horn (Kijas et al., 2012; Pan et al., 2018), pigmentation (Naval-Sanchez et al., 2018), fat deposition (Zhao et al., 2017), tail type (Zhao et al., 2017), wool quality (Fariello et al., 2014), muscle hypertrophy (Fariello et al., 2014), reproduction (Fariello et al., 2014), adaptation to climate (Wei et al., 2016), and resistance to parasites(Fariello et al., 2014), the genetic mechanisms responsible for the numerous evolutionary differences between domestic sheep and their wild ancestors, such as visual deterioration and tame disposition, are still unclear.

To clarify the underlying genes or variants responsible for evolutionary differences and phenotypic changes in domestic sheep, we performed pooled whole-genome resequencing of 338 sheep from five breeds representative of indigenous Chinese breeds and compared these data with publicly available genome sequences of 17 wild sheep (Asiatic mouflon, *Ovis orientalis*). We aimed to elucidate the genetic mechanisms underlying the genetic variation and the phenotypic changes induced in domestic sheep under the influences of artificial and natural selection.

## Materials and Methods

### Ethics Statement

All experiments in this study were carried out in accordance with the approved guidelines of the Regulation of the Standing Committee of Gansu People’s Congress. All experimental protocols and the collection of samples were approved by the Ethics Committee of Gansu Agriculture University under permission no. DK-005.

### Samples and DNA Resequencing

We sampled 338 female sheep (*Ovis aries*) of five geographically and phenotypically representative native Chinese sheep breeds (Supplementary Table S1). Whole-blood samples (5 mL) were collected from 80 Tibetan sheep, 89 Mongolian sheep, 50 Altay sheep, 58 Hu sheep, and 61 Duolang sheep. Genomic DNA was extracted from whole-blood samples according to a standard phenol-chloroform extraction method (Listed, 2001). After dilution to 100 ng/μL, genomic DNA samples from all 338 individuals were mixed in equal volumes to generate 10 pooled libraries (two per breed to reduce sequencing bias, Supplementary Table S1) with a mean insertion size of 500 bp. The libraries were sequenced in 150-bp pair-end reads using a HiSeq XTEN instrument (Illumina, San Diego, CA, United States). In addition, the sequencing data of 17 *Ovis orientalis* individuals were downloaded from National Center for Biotechnology Information (NCBI^1^, Supplementary Table S2) database as a control for *Ovis aries*. FastQC software were used to perform quality control of the raw sequence reads; the index and barcoded sequences were removed, and the unpaired reads were discarded. Subsequently, all clean reads were mapped against the sheep reference genome (Oar_v4.0) using Bowtie2 (Langmead and Salzberg, 2012). First, we used the “bowtie2-built” command line utility to create an index from the Oar_v4.0 reference genome and alignments were prepared using an “end-to-end” strategy. The “-no-mixed” parameter was used to discard the unpaired and multiple alignments for pair-end reads. Alignment files (SAM files) were merged and converted to BAM files prior to sorting and indexing using SAMTOOLS (Li et al., 2009). The potential PCR duplicates were filtered using the “MarkDuplicates” command line of Picard software^2^. The follow-up procedures, including gap realignment and base recalibration, were performed using Genome Analysis Toolkit (GATK, version 3.4) (McKenna et al., 2010).

### Variant Calling

Genomic regions under environmental and artificial selection were identified in domestic sheep by calling the variations from native Chinese sheep breeds and 17 wild sheep (*Ovis orientalis*) using the “UnifiedGenotyper” and “SelectVariants” arguments in GATK (McKenna et al., 2010) and “mpileup-DSuf” arguments in SAMTOOLS (Li et al., 2009) simultaneously. The “concordance” argument of GATK was used to determine the intersection of these two strategies for identification of candidate variations. The SNP filtering criteria were as follows: (1) hard filtration with parameter ‘QD < 20.0 || ReadPosRankSum < -8.0 || FS > 10.0 || QUAL < $MEANQUAL’; (2) “–max-missing 0.7 || –maf 0.05”; (3) filtering of SNPs with a coverage < 8. The Indel filtering criteria were as follows: (1) hard filtration with parameter ‘QUAL < 20.0 || QD < 2.0 || ReadPosRankSum < -8.0 || FS > 10.0 || QUAL < $MEANQUAL; (2) “–maxIndelSize 25 || –maf 0.05”;(3) only insertions and deletions shorter than or equal to 25 bp Indels were taken into account; (4) filtering of Indels with a coverage < 8. The novel SNPs were evaluated by comparison of the SNPs identified using this approach with those of the Build 143 of the NCBI sheep dbSNP database^3^. Except variants summary, only mapped autosomal SNPs were used in the downstream analyses, including selective sweep, gene ontology (GO) and candidate gene analyses. Details of the overall analysis pipeline are shown in Supplementary Figure S1.

### Population Genetics Analyses

For principal component analysis (PCA) analysis, we used genome-wide complex trait analysis (GCTA) to estimate the eigenvectors (Yang et al., 2011), which are asymptotically equivalent to those from PCA implemented using EIGENSTRAT (Price et al., 2006). In this analysis, we incorporated genotype data from five indigenous Chinese sheep breeds (10 pools) and 17 wild sheep (*Ovis orientalis*). A NJ tree was constructed with PLINK (version 1.9) using the matrix of pairwise genetics distances (Chen et al., 2018).

### Analysis of Selective Sweep Regions in Indigenous Chinese Sheep

To explore the genome-wide selective sweep regions in native Chinese sheep, we compared a pool of five indigenous Chinese sheep (*Ovis aries*) with Asian Mouflon (*Ovis orientalis*). Using a sliding-window method (150-kb window with 75-kb sliding steps), the genome-wide *F*_ST_ value distribution was calculated for the following eleven comparisons: *Ovis aries* versus (vs.) *Ovis orientalis*, Altay sheep vs. *Ovis orientalis*, Duolang sheep vs. *Ovis orientalis*, Hu sheep vs. *Ovis orientalis*, Mongolian sheep vs. *Ovis orientalis*, Tibetan sheep vs. *Ovis orientalis*, Altay sheep vs. all other native Chinese sheep, Duolang sheep vs. all other native Chinese sheep, Hu sheep vs. all other native Chinese sheep, Mongolian sheep vs. all other native Chinese sheep and Tibetan sheep vs. all other native Chinese sheep. The homozygosity (*H*_P_) values were calculated based on the number of major and minor allele reads from all SNPs within the 150-kb window with 75-kb sliding steps in each group using the formula described by Wang *et al.* (2015). In the *F*_ST_ analysis, allele frequencies were assessed based on the number of allele reads in native Chinese sheep pools and reliable genotypes in Asian Mouflon. The *F*_ST_ values were then calculated according to the formula described by Weir and Cockerham (1984). To avoid false-positive selection signals, the windows containing fewer than 10 SNPs were discarded. The *F*_ST_ and *H*_P_ values were Z-transformed into *Z*(*F*_ST_) and *Z*(*H*_P_) values according to the formula described by Axelsson et al. (2013). The windows simultaneously containing the top 1% of the high *F_ST_* values and the top 1% of the low *H_P_* value were considered to be regions selected under domestication.

### Candidate Gene Analysis

The identified selection regions were annotated to the closest genes (Oar_v4.0). Genes located in selection regions were identified as candidate genes. Function enrichment of candidate genes including GO categories and HPO (Human Phenotype Ontology) terms were analyzed using g:Profiler^4^ (Reimand et al., 2016). For the target genes, we compared the *F*_ST_ and *H*_P_ values of the target genes with those of their adjacent genomic regions and summarized the allele frequencies of the SNPs in the target genes across native sheep breeds and Asian Mouflon. The PolyPhen Scores of missense mutations were predicted by PolyPhen-2 software (Polymorphism Phenotyping V2)^5^.

## Results and Discussion

### Sequencing, Alignment, and Identification of Single Nucleotide Polymorphisms

Pools of DNA of five indigenous Chinese sheep [Tibetan sheep (*n* = 80), Mongolian sheep (*n* = 89), Altay sheep (*n* = 50), Hu sheep (*n* = 58), and Duolang sheep (*n* = 61) (Supplementary Table S1)] were sequenced at approximately 45× coverage each and then analyzed jointly with publicly available genomes of 17 Asian Mouflon (*Ovis orientalis*, Supplementary Table S2). In total, 9.51 billion reads or 1,427 Gb of genome data were generated in the present study. Stringent quality filtering yielded 8.49 billion reads or 1,172 Gb of genome data (approximately 450× effective sequence depth) for the subsequent analyses (Supplementary Table S3).

**FIGURE 1:**
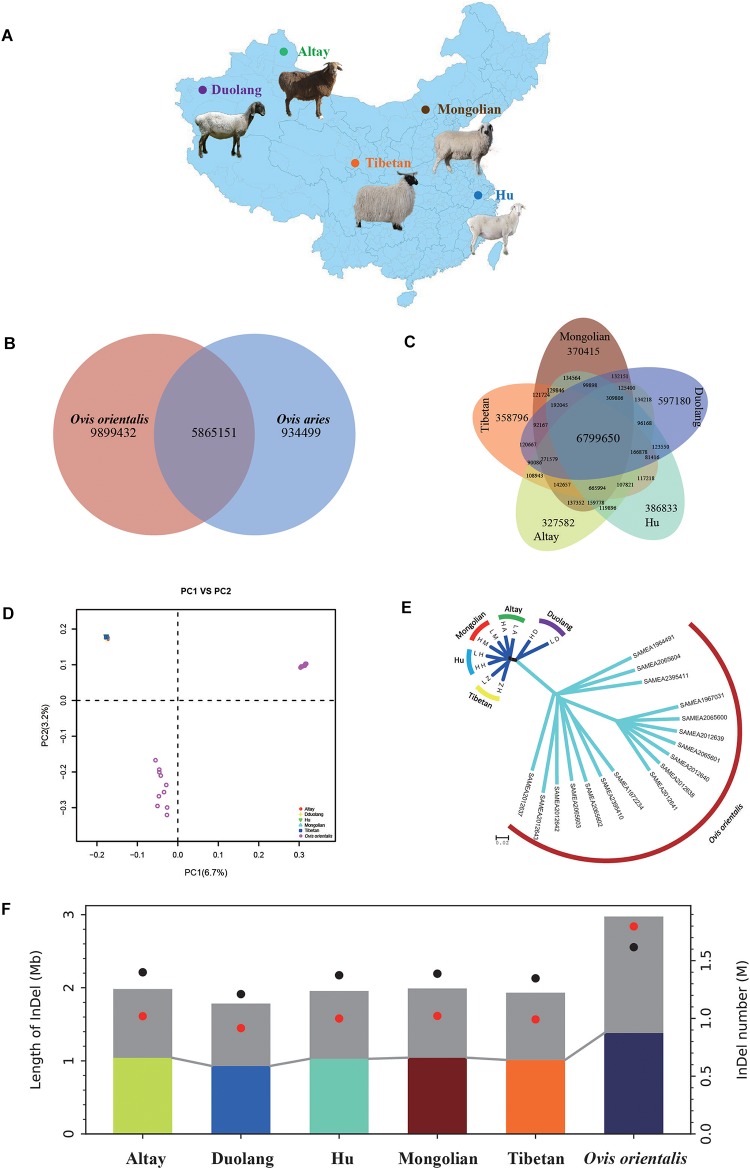
Geographic distribution and population genetics analyses of five indigenous Chinese sheep breeds (*Ovis aries*) and Asian Mouflon (*Ovis orientalis*). **(A)** Geographic distribution of five indigenous Chinese sheep breeds (*n* = 338 sheep). The map was generated uusing Adobe Illustrator CS6 software (https://www.adobe.com/cn/products/illustrator.html). **(B)** Venn diagram showing the shared single nucleotide polymorphisms (SNPs) between *Ovis aries* and *Ovis orientalis*. **(C)** Venn diagram showing the shared SNPs between the five indigenous Chinese sheep breeds. **(D)** Principal component (PC) analysis; PC 1 versus PC 2. **(E)** Neighbor-joining tree. **(F)** The length and number of indels in five indigenous Chinese sheep breeds and *Ovis orientalis*. Black and red dots represent the number of insertions and indels, respectively. Colored and gray pillars represent the length of insertions and indels, respectively.

Using Bowtie2, reads were aligned to the *Ovis aries* reference genome sequence (Oar v4.0) with an average of 97.40% covering 98.77% of the reference genome (Supplementary Table S3). A total of approximately 64 million SNPs were detected and breed-specific SNPs were identified by SAMTOOLS and GATK (Figure 1 and Supplementary Table S4). An average of 93.39% (93.14–93.52%) of the SNPs identified in the five indigenous Chinese sheep breeds were validated by comparison with the sheep dbSNP database (Supplementary Table S4). We obtained 9.89 million SNPs for Altay sheep, 9.57 million for Duolang sheep, 9.76 million for Hu sheep, 9.69 million for Mongolian sheep, 9.43 million for Tibetan sheep, and 15.76 million for *Ovis orientalis*, respectively (Supplementary Tables S4, S5). We identified 5.87 million SNPs shared between *Ovis aries* and *Ovis Orientalis*, as well as 9.90 million and 0.93 million SNPs unique to *Ovis Orientalis* and *Ovis aries*, respectively (Figure 1B and Supplementary Table S5). We found 6.80 million SNPs shared among five indigenous Chinese sheep breeds, as well as 0.33 million, 0.60 million, 0.39 million, 0.37 million, and 0.36 million SNPs unique to the Altay, Duolang, Hu, Mongolian, and Tibetan breeds, respectively (Figure 1C and Supplementary Table S5). The numbers of SNPs in the exonic, intronic, and intergenic regions and chromosomes of each of the five indigenous Chinese sheep breeds were lower than those of *Ovis orientalis* (Supplementary Tables S6, S7). Furthermore, we found higher genomic diversity in the Asian Mouflon than among the five indigenous Chinese sheep breeds, which indicates that the domestic sheep breeds have undergone intense natural and artificial selection.

In addition, an average of 1,220,638 indels (<100 bp) were identified for each of the five indigenous Chinese sheep breeds and 1,882,234 for *Ovis orientalis* (Figure 1F and Supplementary Table S8). Most indels were located in intergenic regions (Supplementary Table S8), which is agreement with the findings of Yang et al. (2016).

### Population Genetics Structure

The PCA (principle component analysis) revealed strong clustering of five native Chinese sheep breeds into a single genetic group, while *Ovis orientalis* was clustered into two groups (Figure 1D). The first two PCs, explaining 6.7 and 3.2% of the total variation, respectively, separated the five native Chinese sheep breeds from *Ovis orientalis*, and *Ovis orientalis* was also divided into two groups with one “*Ovis orientalis*” group at an intermediate position. The neighbor-joining (NJ) tree analysis provided corroborating evidence of these separations, with each breed partitioned into its own clade (Figure 1E). The five native Chinese sheep breeds clustered together, while all 17 *Ovis orientalis* clustered together.

### Genome-Wide Selective Sweep Analysis

**FIGURE 2:**
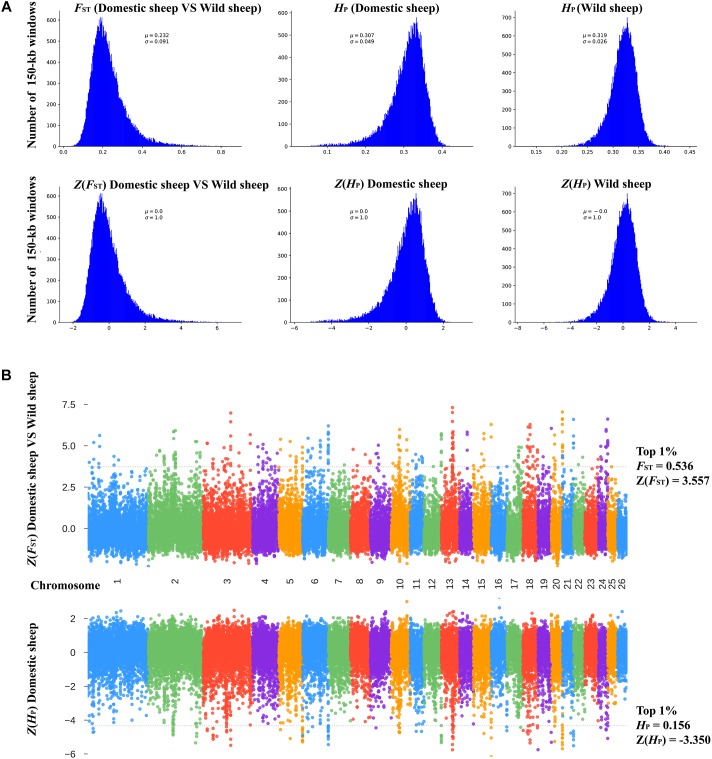
Genome-wide selection analyses identified 98 candidate regions associated with domestication. **(A)** Distribution of Z-transformed average pooled heterozygosity in domestic sheep [*H*_P (Domestic sheep)_ and *Z*(*H*_P_)_Domestic sheep_] and Asian Mouflon [*H*_P (Asian Mouflon)_ and *Z*(*H*_P_)_Asian Mouflon_] as well as average fixation index [*F*_ST(Domestic sheep vs. AsianMouflon)_ and Z(*F*_ST_)_Domestic sheep vs. Asian Mouflon_] values for 150-kb windows (*σ*, standard deviation; *μ*, average). **(B)** Plot of the *Z(H*_P_*)* and *Z(F*_ST_*)* values for domestic sheep along the sheep genome. A dotted line indicates the cut-off [*Z(H*_P_*)* ≤ –3.350 and *Z(F*_ST_*)* ≥ 3.557] used for extracting outliers.

We compared the genomes of five native Chinese sheep breeds with that of Asian Mouflon (*Ovis orientalis*) to identify the domestic sheep genome and signatures of positive selection in response to natural and artificial selection. To accurately detect genomic regions under both selection conditions in domestic sheep, we combined five native Chinese sheep breeds as a whole (domestic sheep) and calculated pooled homozygosity (*H_P_*) and fixation index (*F_ST_*) in the 150-kb windows with 75-kb sliding steps along the autosome. Subsequently, the windows with significantly high *F_ST_* values [top 1%, *F_ST_* > 0.536, *Z*(*F_ST_*) > 3.557] as well as significantly low *H_P_* values [top 1%, *H_P_* < 0.156, *Z*(*H_P_*) < -3.350] were considered as the target windows in domestic sheep (Figure 2B). By applying these standards, we identified 326 regions on autosomes with highly elevated *F_ST_* values (average *F_ST_* = 0.621, range 0.536–0.862, average autosomal *F_ST_* = 0.232, Figure 2A and Supplementary Table S9) and 326 genomic regions in the domestic sheep with extremely low levels of heterozygosity (average *H_P_* = 0.119, range 0.028–0.156, average autosomal *H_P_* = 0.307, Supplementary Table S10). After taking the intersection between these two parameters and merging neighboring windows into the selected regions, 98 putative selective regions with a total length of 23.025 Mb were identified in 26 autosomes, accounting for 0.890% of the entire genome (Supplementary Table S11). These two parameters were then used to identify the putative selective regions in each native Chinese sheep breed. For Altay sheep, 92 putative selective regions (total length, 20.925 Mb; 0.809% of the complete genome) were identified from 26 autosomes (Supplementary Tables S12–14 and Supplementary Figure S2). For Duolang sheep, 95 putative selective regions (total length, 19.875 Mb; 0.768% of the complete genome) were identified from 26 autosomes (Supplementary Tables S15–17 and Supplementary Figure S3). For Hu sheep, 98 putative selective regions (total length, 21.6 Mb; 0.835% of the complete genome) were identified from 26 autosomes (Supplementary Tables S18–20 and Supplementary Figure S4). For Mongolian sheep, 92 putative selective regions (total length, 21.675 Mb; 0.838% of the complete genome) were identified from 26 autosomes (Supplementary Tables S21–23 and Supplementary Figure S5). For Tibetan sheep, 99 putative selective regions (total length, 21.675 Mb; 0.838% of the complete genome) were identified from 26 autosomes (Supplementary Tables S24–26 and Supplementary Figure S6). The selective regions of the native Chinese sheep breeds were intersected to screen for those shared by these five breeds (Supplementary Table S27). This process revealed 18 putative selective regions (30 selective windows, Supplementary Figure S7; total length, 3.6 Mb; 0.139% of the complete genome) were identified from 26 autosomes (Supplementary Table S28). Gene ontology analysis revealed significant enrichment of the 67 candidate genes embedded in the selective regions in 180 GO terms (*P* < 0.05, Supplementary Table S29), including bone morphogenesis (GO terms ID: 0060349), fibroblast growth factor receptor apoptotic signaling pathway (GO terms ID: 1902178), negative regulation of developmental growth (GO terms ID: 0048640), in utero embryonic development (GO terms ID: 0001701), and neural crest cell development (GO terms ID: 0014032). Subsequently, we investigated the general functions of candidate genes involved in decreased vision (*PDE6B*, *RNF24*, and *FOXC1/GMDS*), hypoxia adaptability (*CYP17*), vertebral development (*T* and *HOX* gene cluster) and reproduction (*BMPR-IB*) across the five native Chinese sheep populations (Table 1).

**Table 1:** Summary of major candidate regions identified from the 150-kb windowed heterozygosity (*H*_P_) and fixation index (*F*_ST_) values for each breed comparison (see Supplementary Tables S9–S27 for summary values of all candidate genes).

Gene	Chromosome	Position (Mb)	Association	Candidate SNP position	PolyPhen Scores^1^	Selected breed
*PDE6B*	6	116.7–116.85	Decreased vision	XM_027971363.1:c.C2284A/p.L762M	0.606	All five native breeds
				XM_027971363.1:c.G2994C/p.A982P	0.973	
*PANK2*	13	50.325–50.55		–	–	
*GMDS*/*FOXC1*	20	50.1–50.325		–	–	
*T*	8	87.525–87.825	Short tail	XM_004011450.4:c.G334T/p.G112W	0.998	Altay Duolang
*BMPR-IB*	6	29.175–29.625	Litter size	XM_027970721.1:c.A746G/p.Q249R	0.381	Hu
*RXFP2*	10	29.4–29.55	Absence of horns	–	–	Duolang Hu
*HOX gene cluster*	4	68.7–68.925	Development of axial skeleton	–	–	Mongolian
*CYP17*	22	22.425–22.575	Hypoxia adaptability	NC_019479.2:g.22521501G > T	–	Tibetan
*MSRB3*	3	153.975–154.125	Ear size	–	–	Duolang
*DNAJB5*	2	53.1–53.25	Heat tolerance	–	–	Duolang


*^1^PolyPhen Scores were predicted by PolyPhen-2 software (Polymorphism Phenotyping V2), which indicated possible impact of an amino acid substitution on the structure and function of a protein.*

### Selected Regions Associated With Decreased Vision on Autosomes of Domestic Sheep

**FIGURE 3:**
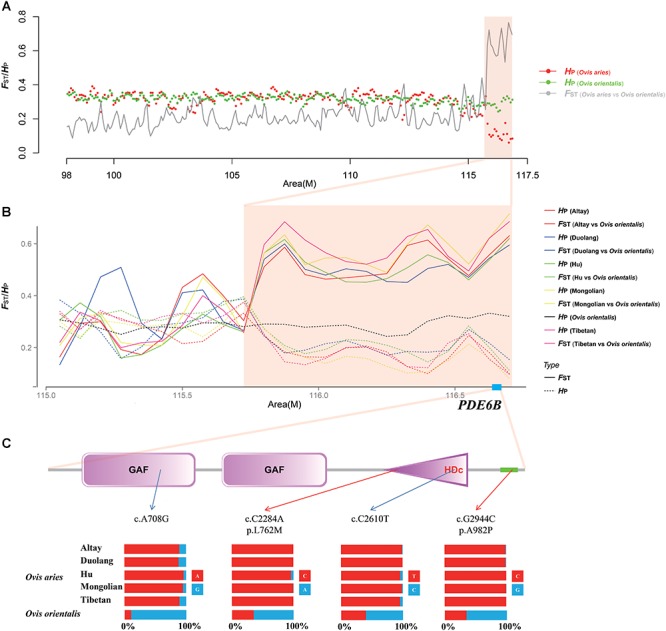
Signatures of the selective sweep of the *PDE6B* gene region in domestic sheep. **(A)** Heterozygosity (*H*_P_) and fixation index (*F*_ST_) values across a chromosome 6 region harboring the *PDE6B* gene. The red and green dots represent the *H*_P_ value of *Ovis aries* and *Ovis orientalis*, respectively. The gray line represents the *F*_ST_ values between *Ovis aries* and *Ovis orientalis*. **(B)** Heterozygosity (*H*_P_) and fixation index (*F*_ST_) values around the *PDE6B* gene region in Asian Mouflon (*Ovis orientalis*) and five indigenous Chinese sheep breeds. Unbroken and dotted lines represent *F*_ST_ and *H*_P_ value, respectively. **(C)** Structure and variations of the *PDE6B* gene. The pink box and triangle indicate the GAF (cGMP-activated PDEs, adenylyl cyclase, and Fh1A) and HDc (HD/PDEase) domains, respectively. The allele frequencies of four SNPs within the *PDE6B* gene across five indigenous Chinese sheep breeds and Asian Mouflon (*Ovis orientalis*).

Vision plays a decisive role in the survival of animals through its influence on a range of core behaviors, such as mating, foraging, and evasion of predators (Yokoyama, 2002). However, domesticated animals such as dogs, horses, ducks, chickens, and sheep, exhibit weaker visual acuity compared to their untamed counterparts (Kruska, 1988; Peichl, 1992; Henderson et al., 2000; Wieser et al., 2013; Fornazari et al., 2016; Wang et al., 2016). The sheep is the one of the most successful and earliest domesticated animal. However, the evolution of vision during sheep domestication has previously received little attention, although the limited findings indicate that domesticated sheep have significantly poorer vision. This change is due to an evolutionary decrease in the regions of the brain involved in processing of visual structures (Kruska, 1988) and eye size corrected for body size (Fornazari et al., 2016). Visual acuity is less important for survival of domestic sheep in the predictable farm envirmonment compared with that required by wild sheep. The four genes in the three shared selected regions of all five native Chinese sheep breeds, phosphodiesterase 6B (*PDE6B*), pantothenate kinase 2 (*PANK2*), and forkhead box C1 (*FOXC1*)/GDP-mannose 4,6-dehydratase (*GDMS*), were enriched for HPO (human phenotype ontology) categories associated with eye development and maintenance as well as vision (Table 2). The *PDE6B* gene encodes the beta subunit of the peripheral membrane heterotrimeric enzyme, cGMP phosphodiesterase (PDE), which a vital protein in the phototransduction pathway (Deterre et al., 1988). *PDE6B* was found to be strongly selected during chicken domestication, and its expression in the brain and retina of domestic chickens was significantly lower than that of their ancestors (Red Junglefowl) (Wang et al., 2016). Mutations in *PDE6B* result in severe retinal diseases including retinitis pigmentosa (McLaughlin et al., 1995), autosomal dominant congenital stationary night blindness (Dryja et al., 1999), and achromatopsia (Gal et al., 1994) in the human, dog, and chicken. In this study, comparison with genomic regions adjacent to the *PDE6B* gene revealed a higher level of population differentiation (*F*_ST_) between domestic sheep and Asian Mouflon (*Ovis orientalis*) and a lower level of nucleotide diversity (*H*_P_) in domestic sheep (Figure 3A,B). These observations indicated the occurrence of a strong selective sweep in the *PDE6B* gene. Further screening of this candidate gene for non-synonymous mutations representing putative functional variants revealed two missense SNPs (c.C2284A/p.L762M and c.G2944C/p.A982P) in Asian Mouflon (*Ovis orientalis*) that are completely fixed in all five domestic sheep breeds. One of these mutations (c.G2994C/p.A982P) is located in the PDE6B protein HDc domain (HD/PDEase domain, IPR003607), which starts at position 706 and ends at position 884 (Figure 3C). The HDc domain represents a related PDE domain that is found in eukaryotic 3′,5′-cGMP PDEs, which are located in the outer segments of the photoreceptor and are light-activated, playing a pivotal role in signal transduction (Muradov et al., 2010). We also detected two synonymous mutations in the HDc domain (c.C2610T) and GAF domain (c.A708G, position 71 and ends at position 310) (Figure 3C). GAF (cGMP-activated PDEs, adenylyl cyclase, and Fh1A) domain, which is characterized by a conserved NKFDE amino acid motif, is necessary for the binding of cyclic nucleotides in PDEs that regulate catalytic activity (Zoraghi et al., 2004). These results suggest that variations in the GAF and HDc domains of *PDE6B* may be associated with the evolution of vision in domestic sheep. Further evaluation and understanding of how the *PDE6B* gene regulates the visual system of sheep will be important in understanding a possible role of *PDE6B* in sheep domestication.

**Table 2:** Human phenotype ontology (HPO) categories of the candidate genes related to vision function.

Chromosome	Position (Mb)	Genes	Human phenotype ontology
6	116.7–116.85	*PDE6B*	Abnormality of the eye (HP:0000478)
			Abnormal eye physiology ( HP:0012373)
			Severe myopia ( HP:0011003)
			Glaucoma ( HP:0000501)
			Abnormal visual electrophysiology (HP:0030453)
			Abnormality of vision (HP:0000504)
			Reduced visual acuity (HP:0007663)
13	50.325–50.55	*PANK2*	Abnormality of the eye (HP:0000478)
			Abnormal eye morphology (HP:0012372)
			Abnormality of the retina (HP:0000479)
			Abnormality of the optic nerve (HP:0000587)
			Abnormality of the optic disk (HP:0012795)
			Optic atrophy (HP:0000648)
20	50.1–50.325	*FOXC1/GMDS*	Abnormality of the eye (HP:0000478)
			Abnormal eye physiology (HP:0012373)
			Abnormality of vision (HP:0000504)
			Visual loss (HP:0000572)
			Glaucoma (HP:0000501)
			Abnormal eye morphology (HP:0012372)
			Aplasia/Hypoplasia of the retina (HP:0008061)
			Ectopia pupillae ( HP:0009918)


Moreover, *PANK2* gene showed the strongest selection signal among the world’s modern sheep breeds (Naval-Sanchez et al., 2018). The *PANK2* gene encodes a pantothenate kinase family protein that is a key regulatory enzyme in coenzyme A biosynthesis (Kuo et al., 2005). *PANK2* mutations are associated with pantothenate kinase-associated neurodegeneration, the major symptoms of which include optic atrophy (Hundallah and Al Hakeem, 2017). Axenovich et al. (2011) found that variants in the *RNF24*/*PANK2* region were significantly associated with changes in optic disk morphology in humans (Axenovich et al., 2011). The *FOXC1* gene belongs to forkhead family of transcription factors characterized by a distinct DNA-binding forkhead domain. These transcription factors are involved in regulating embryonic and ocular development and *FOXC1* mutations are associated with various glaucoma phenotypes, including primary congenital glaucoma and Axenfeld–Rieger syndrome, which characterized by specific ocular anomalies (Nishimura et al., 1998). Mutations in in the *GMDS* gene, which encodes a protein that is essential for the first step in synthesis of GDP-fucose from GDP-mannose, have also been shown to be associated with POAG (primary open-angle glaucoma), a disorder characterized by chronic neurodegenerative optic neuropathy (Gharahkhani et al., 2014; Wiggs and Pasquale, 2017). In this study, we detected two strong selection signals in *PANK2* [*F*_ST__(_*_Ovis aries_*_vs._ *_Ovis orientalis_*_)_ = 0.86, *H*_P(_*_Ovis aries_*_)_ = 0.05, Supplementary Figures S8a,b), and *FOXC1/GDMS* [*F*_ST__(_*_Ovis aries_*_vs._ *_Ovis orientalis_*_)_ = 0.84, *H*_P(_*_Ovis aries_*_)_ = 0.05, Supplementary Figures S9a,b) in all five native sheep breeds compared with the Asian Mouflon (*Ovis orientalis*). These results indicate that the strong selection signals in these genes may cause the optic atrophy and ocular anomalies and decreased vision in domestic sheep.

### Adaptation of Tibetan Sheep Living in the Qinghai-Tibet Plateau to Hypoxia Challenges

**FIGURE 4:**
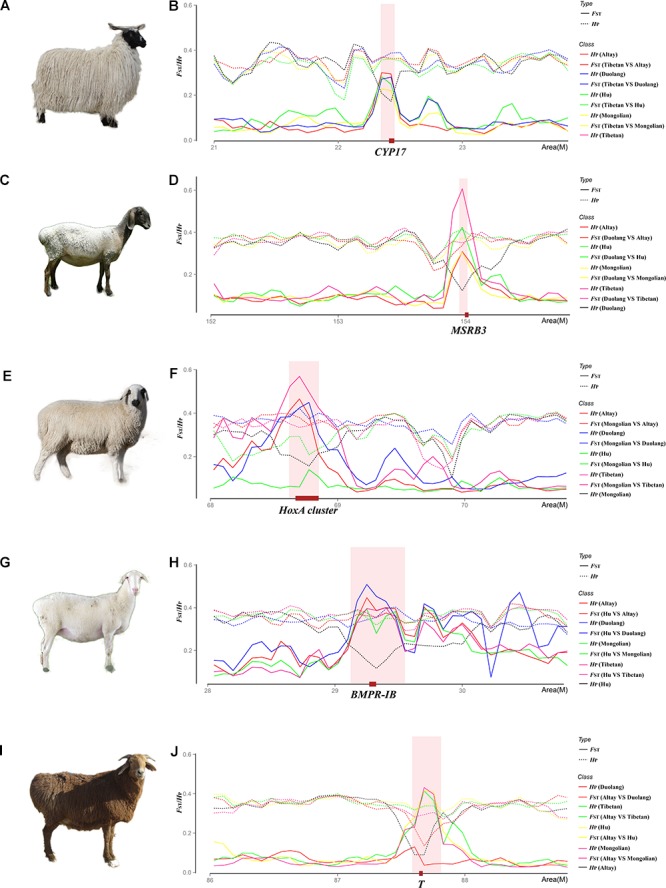
Specific selection regions in each indigenous Chinese sheep breed. **(A)** Image of Tibetan sheep. **(B)** Signatures of the selective sweep of the *CYP17* gene region associated with hypoxia adaptability in Tibetan sheep. **(C)** Image of Duolang sheep. **(D)** Signatures of the selective sweep of the *MSRB3* gene region associated with ear size in Duolang sheep. **(E)** Image of Mongolian sheep. **(F)** Signatures of the selective sweep of the *HoxA* gene cluster region associated with vertebral number variations in Mongolian sheep. **(G)** Image of Hu sheep. **(H)** Signatures of the selective sweep of the *BMPR-IB* gene region associated with litter size in Hu sheep. **(I)** Image of Altay sheep. **(J)** Signatures of the selective sweep of the *T* gene region associated with the short-tailed phenotype in Altay sheep.

Low oxygen is one of the most severe environmental challenges for animals living in high-altitude regions, such as the Qinghai-Tibet Plateau, where the average altitude exceeds 4,000 m. The Tibetan sheep, a native Chinese sheep breed, adapts well to the extreme high-altitude stress of its environment (Yang et al., 2016) through responses that critically involve the suppressor of cytokine signaling 2 (*SOCS2*) gene (Wei et al., 2016; Yang et al., 2016). In this study, comparison with genomic regions adjacent to the *SOCS2* gene showed a higher level population differentiation (*F*_ST_ = 0.628, Supplementary Table S23) between Tibetan sheep and Asian Mouflon (*Ovis orientalis*) and lower level of nucleotide diversity (*H*_P_ = 0.180, Supplementary Table S24) in Tibetan sheep. These observations indicate that a strong selective sweep in the *SOCS2* gene is responsible for hypoxia adaptability during domestication of Tibetan sheep. More strikingly, we found that the *CYP17* (cytochrome P-450 17alpha-hydroxylase/C17, 20-lyase) gene was strongly selected in Tibetan sheep compared to the Asian Mouflon [*F*_ST(Tibetan sheep_ _vs._ *_Ovis orientalis_*_)_ = 0.628, *H*_P (Tibetan sheep)_ = 0.173, Supplementary Tables S23, S24] and other four native Chinese sheep breeds [*F*_ST (Tibetan sheep_ _vs._ _Altay sheep)_ = 0.322, *F*_ST (Tibetan sheep_ _vs._ _Duolang sheep)_ = 0.280, *F*_ST (Tibetan sheep_ _vs._ _Hu sheep)_ = 0.249, *F*_ST (Tibetan sheep vs._ _Mongolian sheep)_ = 0.221, Figure 4A,B). The *CYP17* gene encodes a CYP subfamily 17A enzyme that catalyzes reactions involved in the synthesis of sex steroid hormones (Gilep et al., 2011), including testosterone, estrogens, and progestins (Marino et al., 2011). These hormones regulate the concentration of hemoglobin levels and red blood mass by affecting the Na-K-ATPase in brain (Sunny and Oommen, 2004) and the frequency of ventilation (Favier et al., 1997; Gonzales et al., 2009). For example, testosterone has been found to decrease ventilation (Favier et al., 1997) and increases erythropoiesis (Gonzales et al., 2009), whereas estrogens have opposing actions (Cristancho et al., 2007; Horiguchi et al., 2005). *CYP17* gene mutations are associated with hemoglobin levels and correlate significantly with high-altitude polycythemia (HAPC) risk (Wu, 2005; Jiang et al., 2014; Xu et al., 2015). In this study, we detected a single SNP (Chr22: 22521501) located downstream (431 bp) of the *CYP17* gene, which is completely corrected in Tibetan sheep (Supplementary Figure S10). The dominant allele frequency of this candidate gene has a tendency to increase with the altitude of the habitats of the five Chinese native sheep breeds. These results suggest that *CYP17* gene mutations confer a selective advantage linked to the hypoxia adaptability in Tibetan sheep through regulation of hemoglobin levels and red cell mass.

### Selective Regions Reflecting Varietal Characteristic in the Other Four Native Chinese Sheep Breeds

Native Chinese sheep breeds are a valuable genetic resource, exhibiting rich phenotypic diversity including morphological and production traits (Zhao et al., 2017). Based on kinship, Chinese-domesticated sheep populations can be grouped into three lineages: Mongolian, Tibetan, and Kazak (Guo et al., 2005). The five Chinese native sheep breeds used in this study are widely distributed and have their own characteristics. As previously noted for example, Tibetan sheep in the Qinghai-Tibet Plateau (average altitude ≥ 4,000 m) adapts well to the low oxygen, low pressure, and high altitude plateau environment (Yang et al., 2016). The Mongolian lineage is also one of the most widely distributed species in China and comprises a variety of sheep breeds such as Mongolian sheep and Hu sheep, with different phenotypes under the different natural conditions or artificial selection (Guo et al., 2005). A strong selective signal in the genome of Mongolian sheep was mapped to the *HoxA* gene cluster region including nine *HoxA* genes (*HoxA1*–*5*, *9–11*, and *13* (Figure 4E,F). The *HoxA* gene cluster is critically involved in axial skeleton development and the expression patterns of these genes are associated with evolutionary changes in the vertebral column (Bohmer and Werneburg, 2017; Burke et al., 1995; Mansfield and Abzhanov, 2010; Bohmer et al., 2015). Thus, selection for this gene has a potential association with vertebral number variations of Mongolian sheep (Zhang et al., 1998). Hu sheep, also belonging to the Mongolian lineage, are a well-known and prolific native Chinese breed (Wang et al., 2018). As expected, strong selective signals in Hu sheep were mapped to the *BMPR-IB* (Figure 4G,H) and *RXFP2* genes, which are associated with major characteristics of Hu sheep, such as prolificacy (Wang et al., 2018) and the absence of horns (Kijas et al., 2012; Pan et al., 2018). *RXFP2* has also been identified in Duolang sheep, which is also a polled breed produced by crossing Jill Wagner sheep from Afghanistan with native Chinese sheep breeds (Wei et al., 2015). Duolang sheep, which show a large genetic distance from other Chinese native sheep breeds, are found in Markit County, Xinjiang, in the western region of the Tarim Basin and exhibit good stress and heat tolerance (Wei et al., 2015). We found five specific selective regions in Duolang sheep compared to Asian Mouflon and the other four native sheep breeds. These regions included 10 genes, such as the DnaJ heat shock protein family (Hsp40) member B5 (*DNAJB5*) and methionine sulfoxide reductase B3 (*MSRB3*) genes (Figure 4C,D). The *DNAJB5* gene encodes a member of the DNAJ heat shock protein 40 family of co-chaperone proteins (Lampis et al., 2018). It plays a vital role in the stress tolerance of immune cells, especially against heat stress (Vjestica et al., 2013; Foster et al., 2015), with molecular chaperone and anti-apoptosis effects in the maintenance of immune cell survival and internal stability (Morimoto, 1993); thus, it is possible that selection for this gene may be associated with heat tolerance in Duolang sheep. Numerous studies indicate that the *MSRB3* gene is also a candidate gene associated with ear size in sheep (Wei et al., 2015), pigs (Zhang et al., 2014), and dog (Boyko et al., 2010). Altay sheep, belonging to the Kazak lineage, inhabit the Altay area (Xinjiang, China) where the winter is long and cold (Li et al., 2018). In this harsh environment, the short-tailed phenotypes of Altay sheep store large amounts of fat on their buttocks as overwinter reserves (Pourlis, 2011). Duolang sheep also exhibit the short-tailed phenotype consistent for the same reason. The short-tailed phenotype is the result of artificial and natural selection favoring a specific set of genetic mutations (Zhi et al., 2018). Strong selective signals were detected in regions of Chr8:87675001-87900000 in Duolang sheep and Chr8:87525000-87825000 in Altay sheep, respectively, compared to Asian Mouflon and other native sheep breeds (Figure 4I,J). The *T* (Brachyury transcription factor) gene harbored in this region was recently linked with the short-tailed phenotype of Hulunbuir sheep (Zhi et al., 2018) and displayed strong evidence for an association with the Brachyury protein in mice (Wu et al., 2010; Pennimpede et al., 2012). The values of pairwise *F*_ST_ between short-tailed sheep breeds (Altay sheep and Duolang sheep) and non-short-tailed sheep breeds (Tibetan sheep, Hu sheep, and Mongolian sheep) were high, while the *F*_ST_ signals were absent in comparisons between short-tailed sheep breeds or non-short-tailed sheep breeds. Furthermore, we found that the c.G334T/p.G112W mutation was almost completely fixed in Altay sheep and Duolang sheep (Supplementary Figure S11), which is consistent with the study in Hulunbuir sheep reported by Zhi et al. (2018). These results suggest that the c.G334T mutation in the *T* gene also contributes to the short-tailed phenotype in Altay sheep and Duolang sheep. Overall, extensive selective signatures detected in this study further elucidate the genetics of diverse phenotypes and the physiological changes in response to long-term artificial and natural selection during sheep domestication.

## Conclusion

In this study, we identified a novel series of vision-related genes and function mutations subjected to positive selection in all five Chinese native sheep breeds during sheep domestication. These genes included *PDE6B* (c.G2994C/p.A982P mutation), *PANK2*, and *FOXC1/GMDS*, that may be responsible for decreased vision during sheep domestication. In addition, some specific selective genes that reflect the characteristics of each breed were also identified in the five native Chinese sheep breeds. These included two novel genes related to hypoxia adaptability (*CYP17*) in Tibetan sheep and heat tolerance (*DNAJB5*) in Duolang sheep, and some known genes associated with the short-tailed phenotype (*T*), the presence or absence of horns (*RXFP2*), vertebral number variations (*HoxA* gene cluster), and litter size (*BMPR-IB*). These results provide new insights into the molecular mechanism of sheep domestication and evolution as well as the formation of the unique characteristics of Chinese native sheep breeds.

## Data Availability

The five Chinese native sheep breeds Bioprojects are accessible at NCBI Bioproject (http://www.ncbi.nlm.nih.gov/bioproject) under accession numbers PRJNA433439. The sequencing data are available from Sequence Read Archive (SRA) database with NCBI-SRA accession numbers SRR6960913–SRR6960922.

## Author Contributions

WW, FL, ZJ, and BL conceived the study. WW, FL, XiaoZ, and YL contributed to sample collection and prepared biological samples. WW, ZJ, YaZ, YuZ, XiaoZ, XiangZ, and CL analyzed the data. YouZ took the photos of five native sheep breeds. WW wrote the manuscript. WW, BL, FL, and ZJ revised the manuscript. All authors read and approved the final manuscript.

## Conflict of Interest Statement

The authors declare that the research was conducted in the absence of any commercial or financial relationships that could be construed as a potential conflict of interest.

## Footnotes

1 https://www.ncbi.nlm.nih.gov/biosample

2 http://broadinstitute.github.io/picard/

3 https://www.ncbi.nlm.nih.gov/snp

4 http://biit.cs.ut.ee/gprofiler/

5 http://genetics.bwh.harvard.edu/pph2/index.shtml
