# Supplementary material for: Deep Genome Resequencing Reveals Artificial and Natural Selection for Visual Deterioration, Plateau Adaptability and High Prolificacy in Chinese Domestic Sheep
Source: Front Genet. 2019 Apr 2;10:300. doi: 10.3389/fgene.2019.00300 (PMC6454055; doi:10.3389/fgene.2019.00300)
Supplement: Supplementary file 1 [file Table_1.DOCX]

**Deep genome resequencing reveals artificial and natural selection for visual deterioration, plateau adaptability and high prolificacy in Chinese domestic sheep**

Weimin Wang^1^, Xiaoxue Zhang^1^, Xiang Zhou^3,4^, Yangzi Zhang^4^, Yongfu La^1^, Yu Zhang^3^, Chong Li^1^, Youzhang Zhao^1^, Fadi Li^1*†,2,5^, Bang Liu^3*†^, Zhihua Jiang^4*†^

**Supplementary Figures**

**Figure S1………………………………………………………………. 2**

**Figure S2………………………………………………………………. 3**

**Figure S3………………………………………………………………. 4**

**Figure S4………………………………………………………………. 5**

**Figure S5………………………………………………………………. 6**

**Figure S6………………………………………………………………. 7**

**Figure S7………………………………………………………………. 8**

**Figure S8………………………………………………………………. 9**

**Figure S9………………………………………………………………. 10**

**Figure S10………………………………………………………………11**

**Figure S11……………………………………………………………….12**


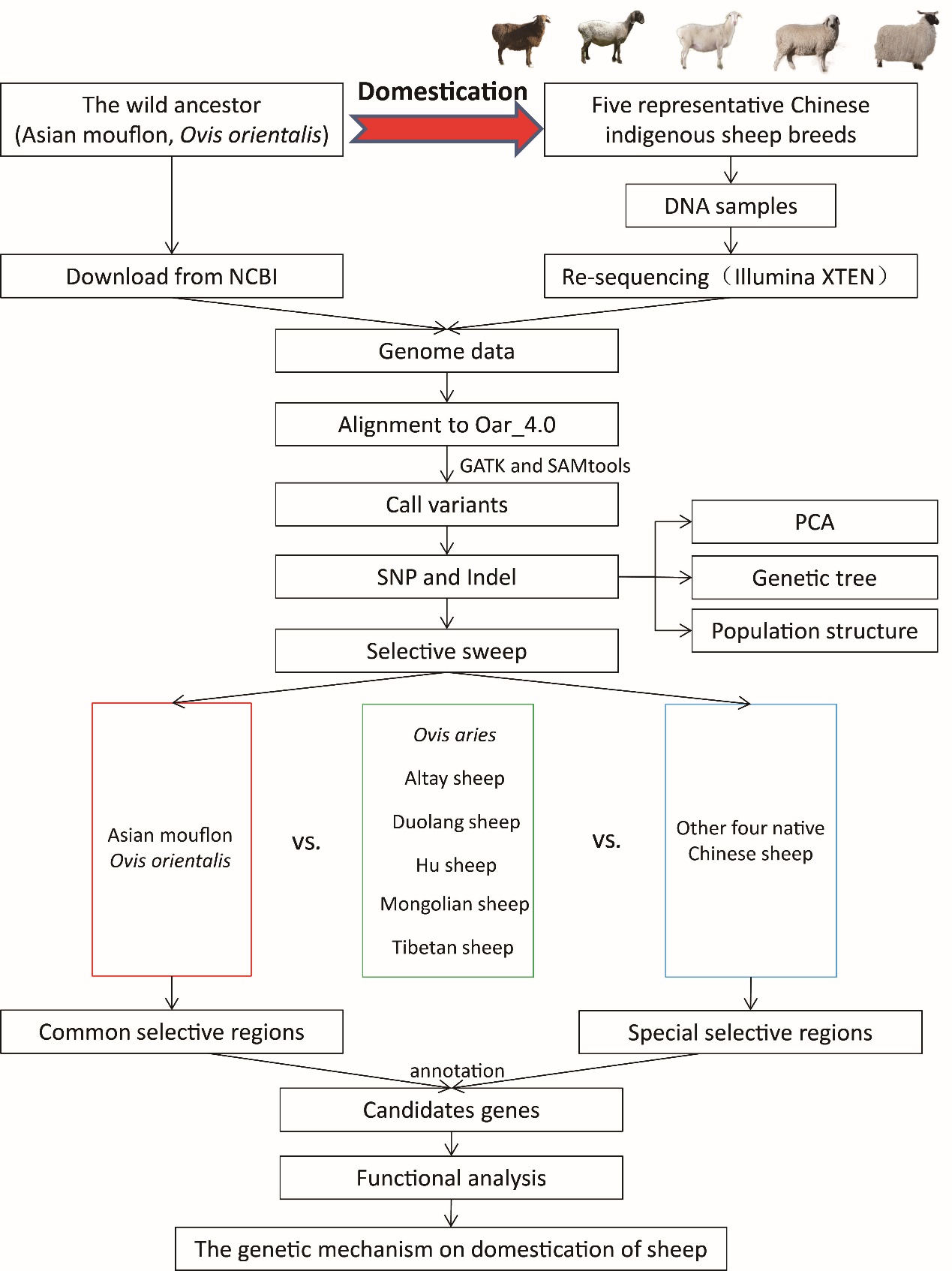


**Figure. S1** Technical route and analysis pipeline.


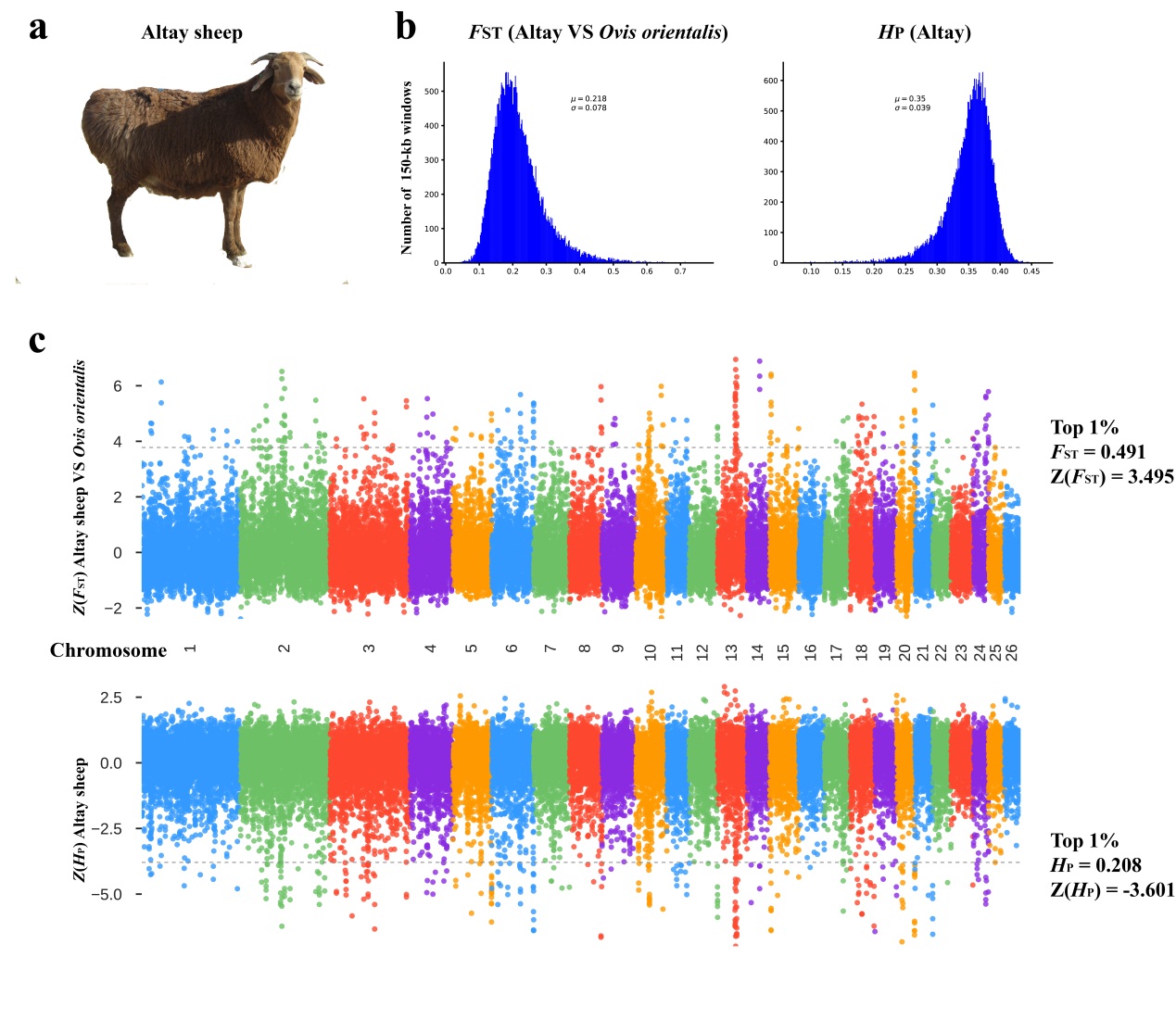


**Figure. S2** Genome-wide selection analysis of Altay sheep. (A) Image of Altay sheep. (B) Histograms of 150-kb windowed heterozygosity (*H*_P_) and fixation index (*F*_ST_) of the autosomes. (C) Plot of the *Z*(*H*_P_) and *Z*(*F*_ST_) values for the Altay sheep along the autosomes.


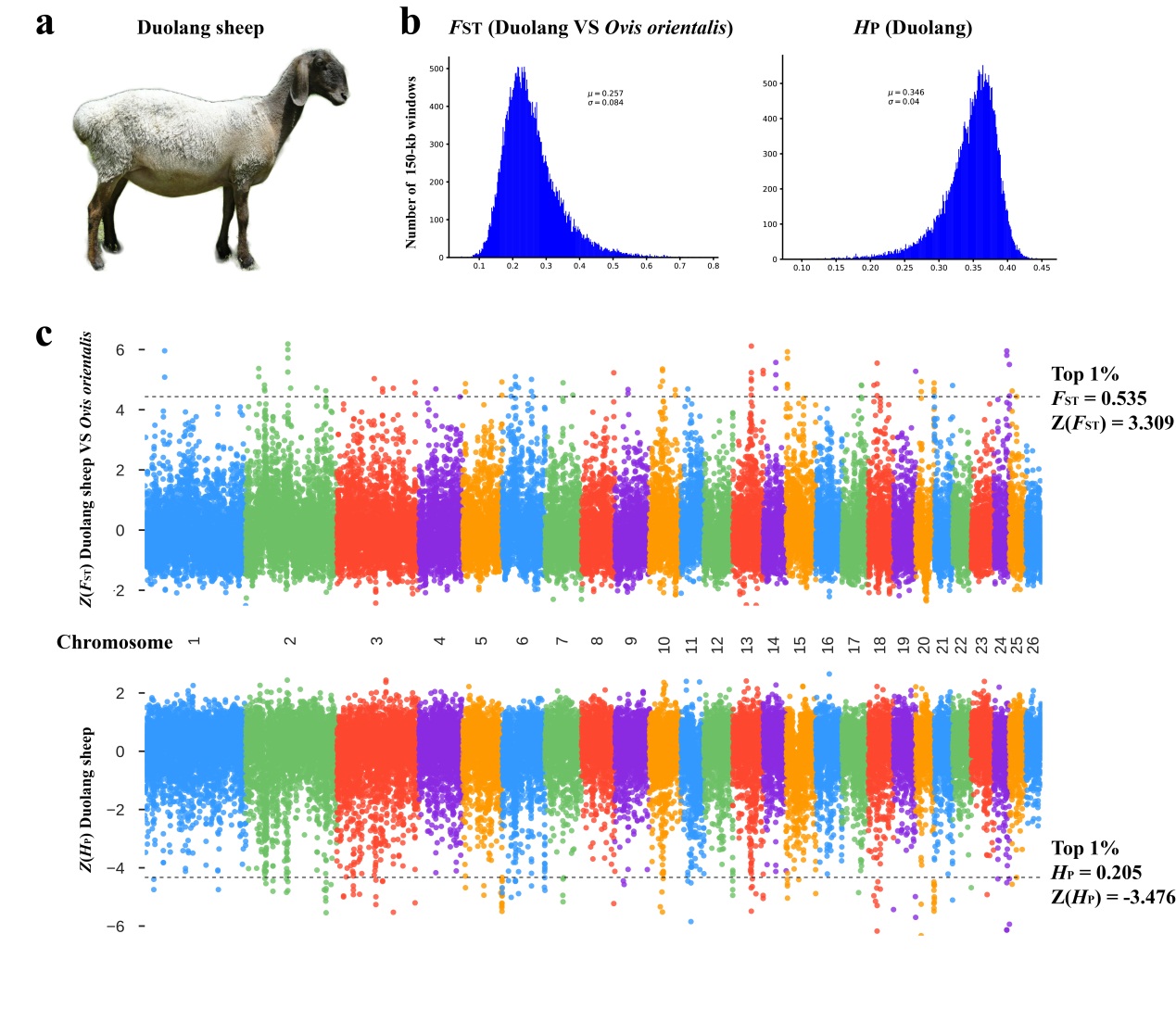


**Figure. S3** Genome-wide selection analysis of Duolang sheep. (A) Image of Duolang sheep. (B) Histograms of 150-kb windowed heterozygosity (*H*_P_) and fixation index (*F*_ST_) of the autosomes. (C) Plot of the *Z*(*H*_P_) and *Z*(*F*_ST_) values for the Duolang sheep along the autosomes.


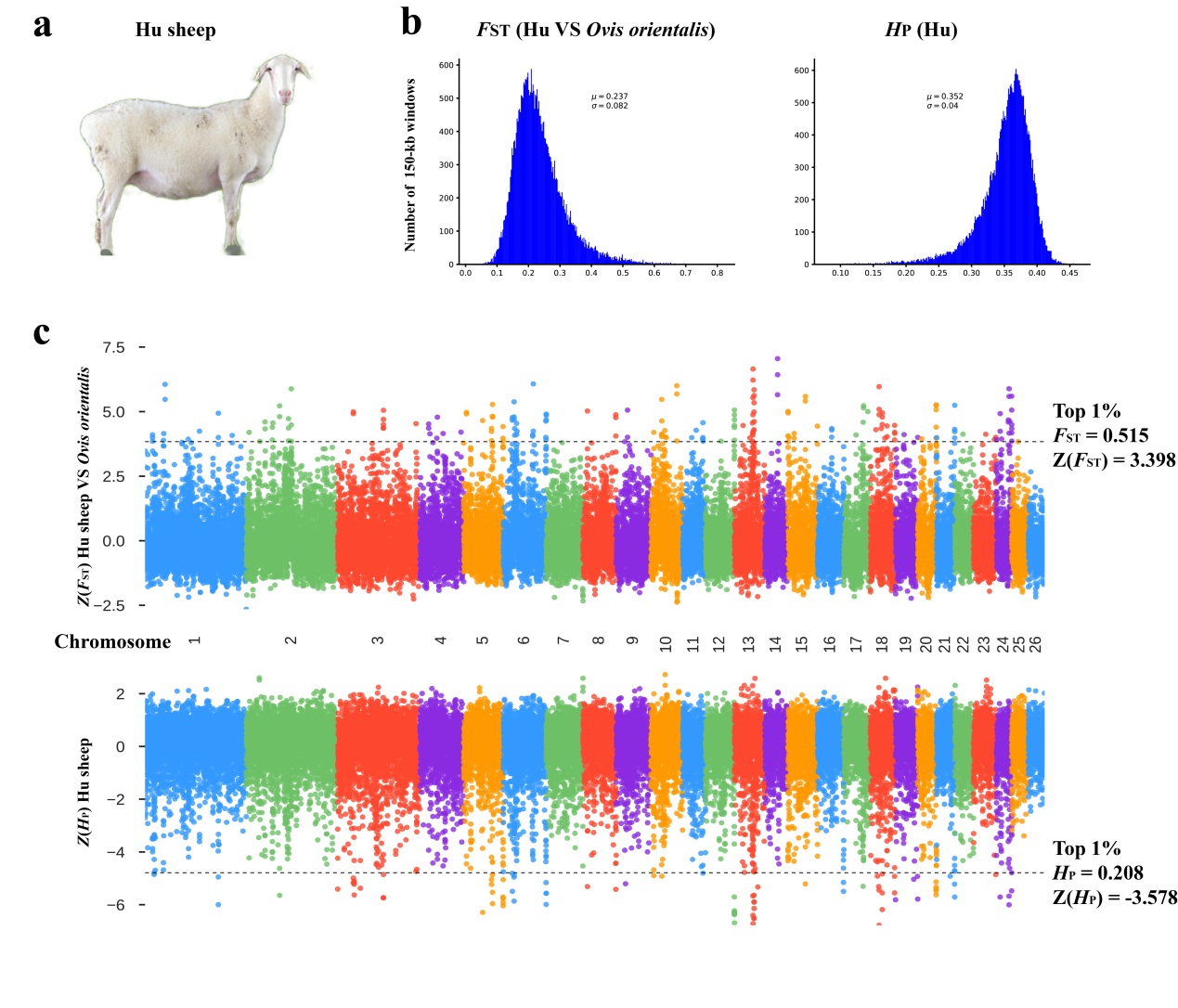


**Figure. S4** Genome-wide selection analysis of Hu sheep. (A) Image of Hu sheep. (B) Histograms of 150-kb windowed heterozygosity (*H*_P_) and fixation index (*F*_ST_) of the autosomes. (C) Plot of the *Z*(*H*_P_) and *Z*(*F*_ST_) values for the Hu sheep along the autosomes.


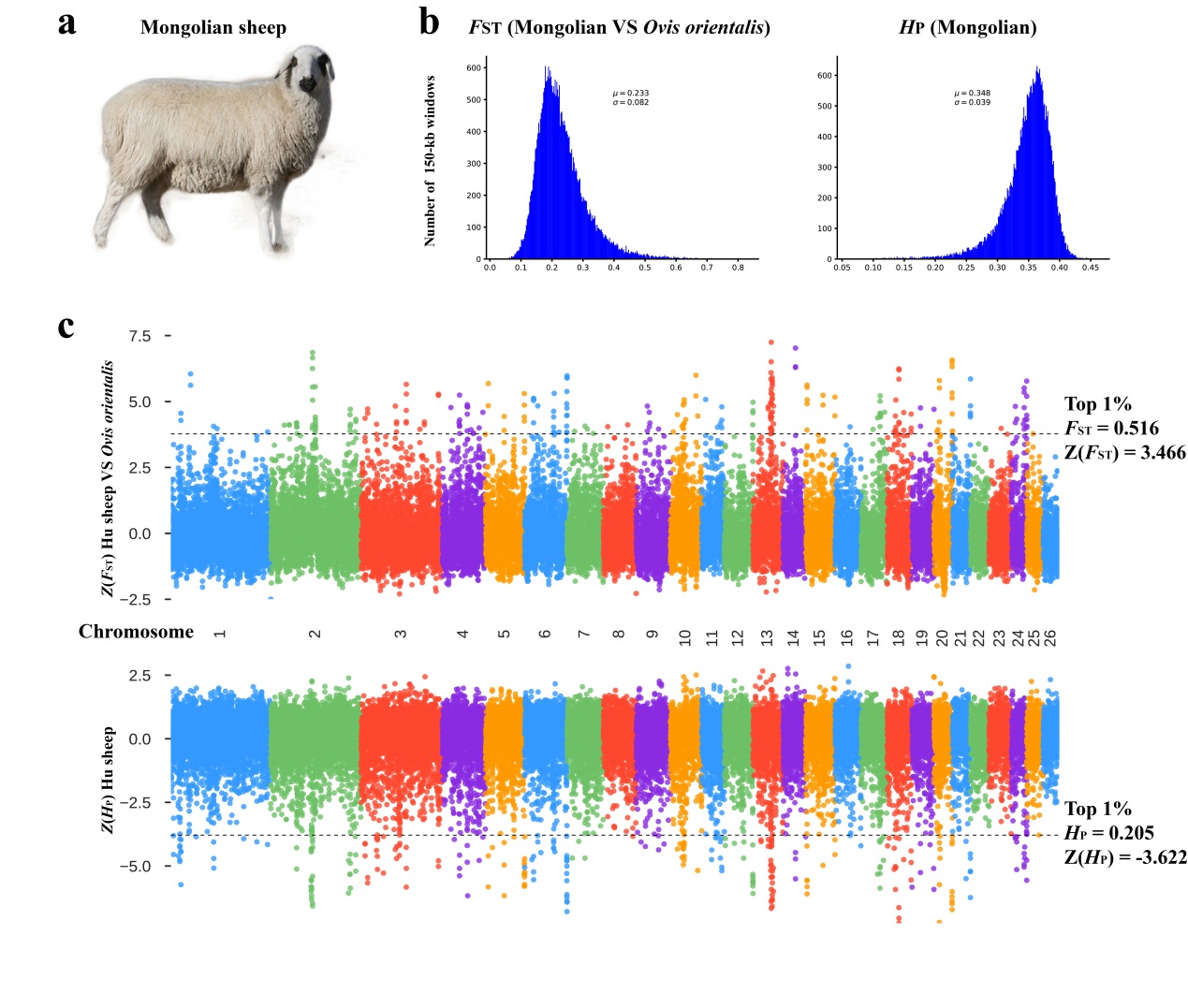


**Figure. S5** Genome-wide selection analysis of Mongolian sheep. (A) Image of Mongolian sheep. (B) Histograms of 150-kb windowed heterozygosity (*H*_P_) and fixation index (*F*_ST_) of the autosomes. (C) Plot of the *Z*(*H*_P_) and *Z*(*F*_ST_) values for the Mongolian sheep along the autosomes.


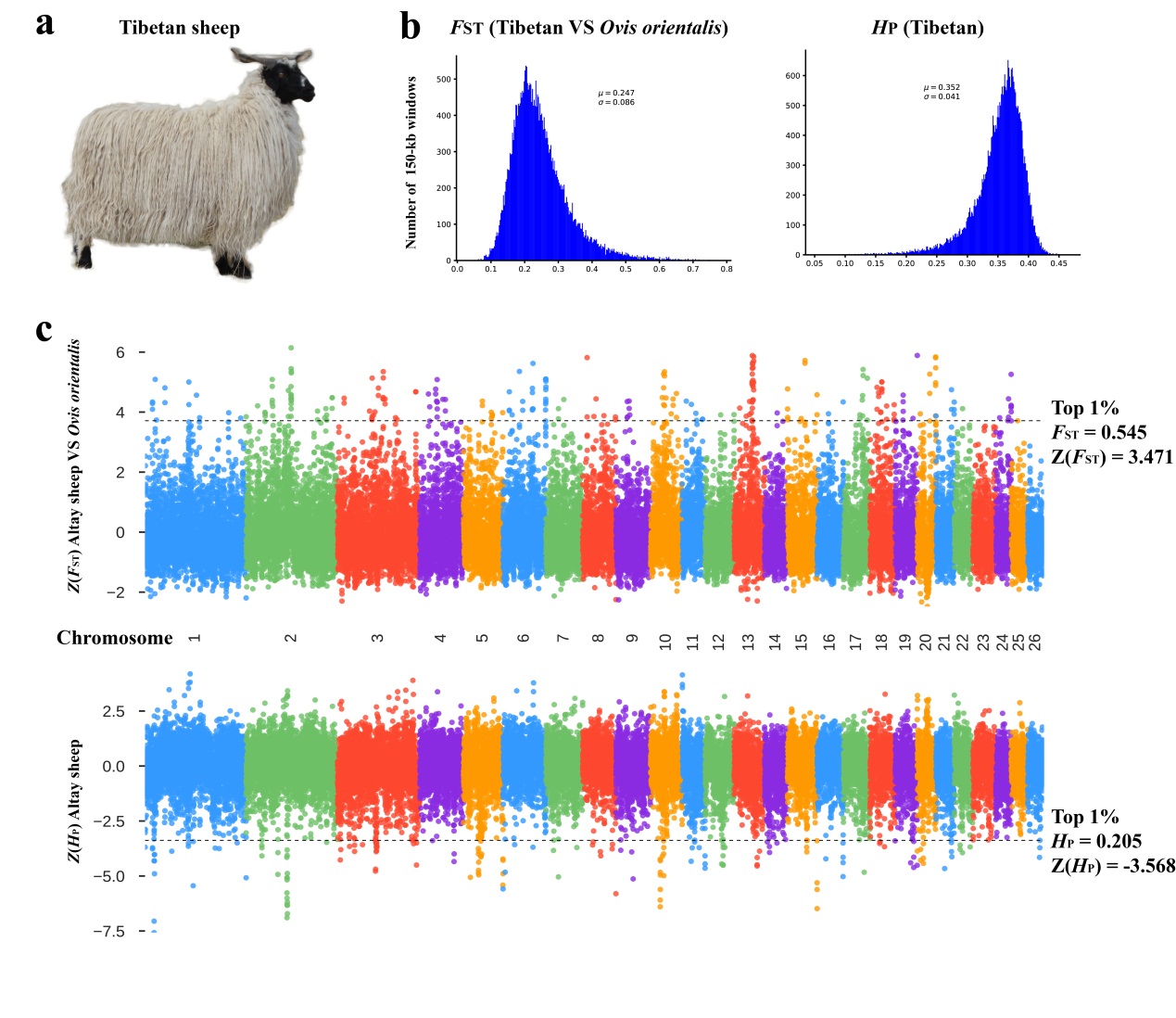


**Figure. S6** Genome-wide selection analysis of Tibetan sheep. (A) Image of Tibetan sheep. (B) Histograms of 150-kb windowed heterozygosity (*H*_P_) and fixation index (*F*_ST_) of the autosomes. (C) Plot of the *Z*(*H*_P_) and *Z*(*F*_ST_) values for the Tibetan sheep along the autosomes.


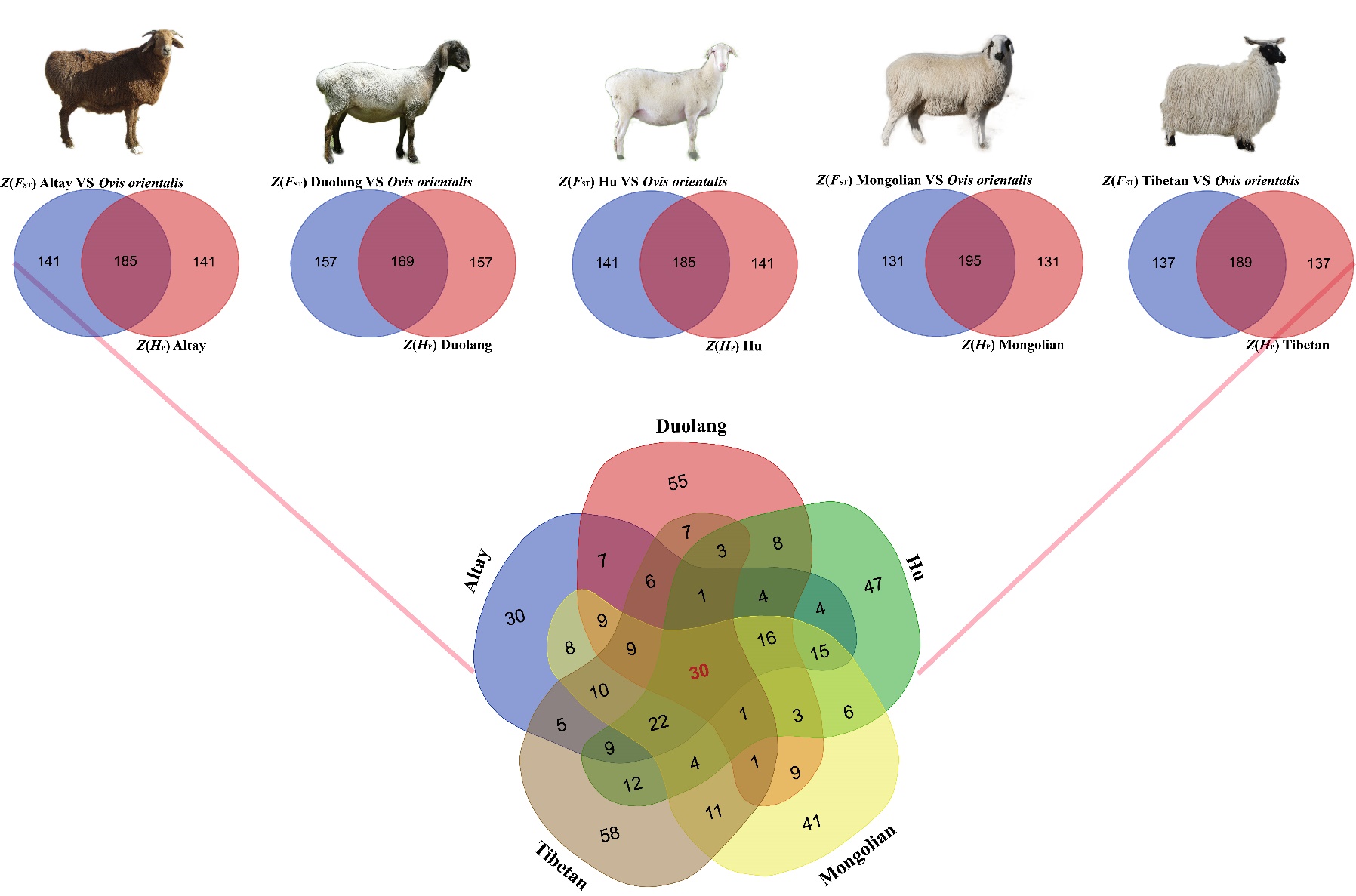


**Figure. S7** Venn diagram of common selected windows among five native Chinese sheep breeds.


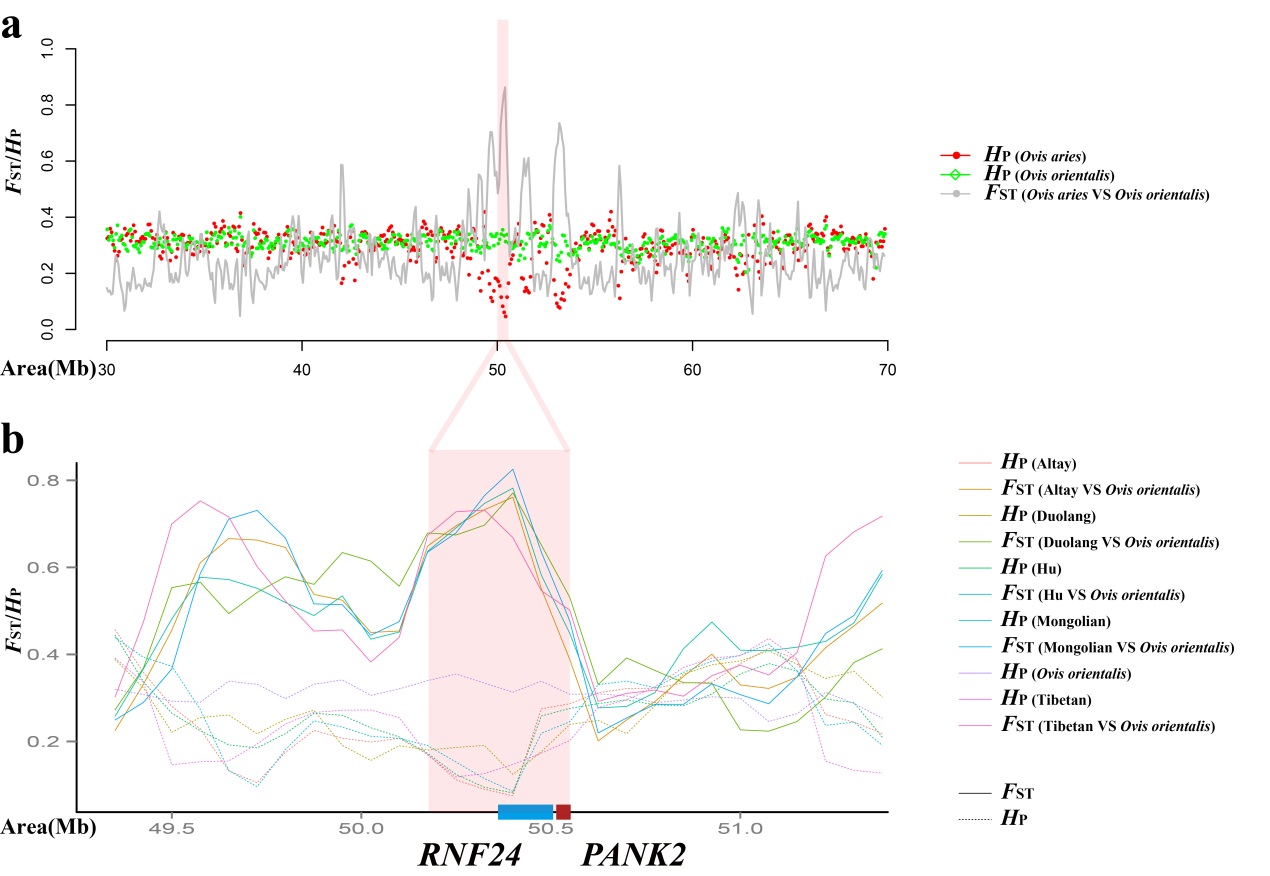


**Figure. S8** Signatures of the selective sweep of the *RNF24/PANK2* gene region in domestic sheep. (A) Heterozygosity (*H*_P_) and fixation index (*F*_ST_) values across a chromosome 13 region harbouring the *PANK2* gene. (B) Heterozygosity (*H*_P_) and fixation index (*F*_ST_) values around the *PANK2* gene region in wild sheep (*Ovis orientalis*) and five native Chinese sheep breeds. Unbroken and dotted lines represent *F*_ST_ and *H*_P_ value, respectively.


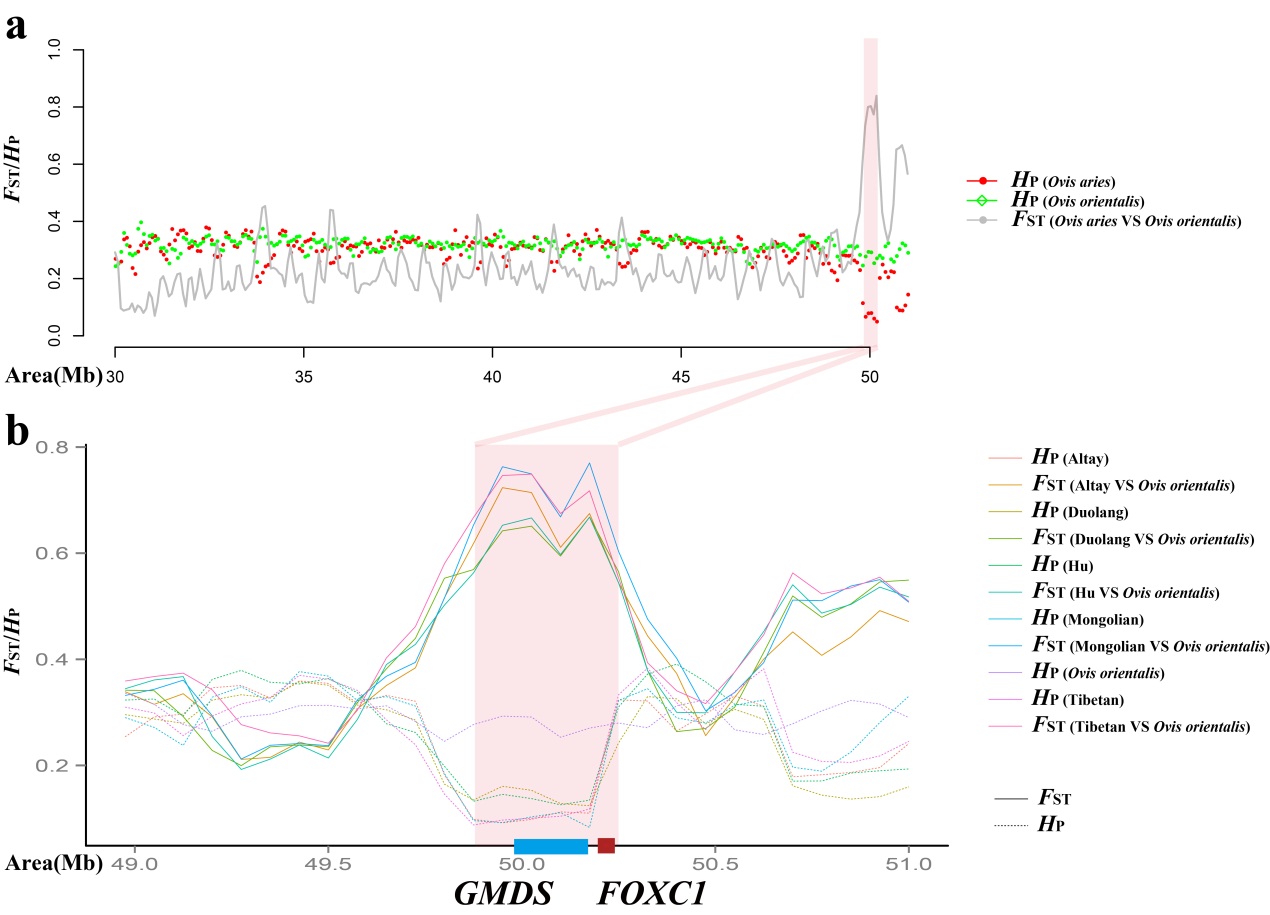


**Figure. S9** Signatures of the selective sweep of the *GMDS/FOXC1* gene region in domestic sheep. (A) Heterozygosity (*H*_P_) and fixation index (*F*_ST_) values across a chromosome 20 region harbouring the *FOXC1* gene. (B) Heterozygosity (*H*_P_) and fixation index (*F*_ST_) values around the *FOXC1* gene region in wild sheep (*Ovis orientalis*) and five native Chinese sheep breeds. Unbroken and dotted lines represent *F*_ST_ and *H*_P_ value, respectively.


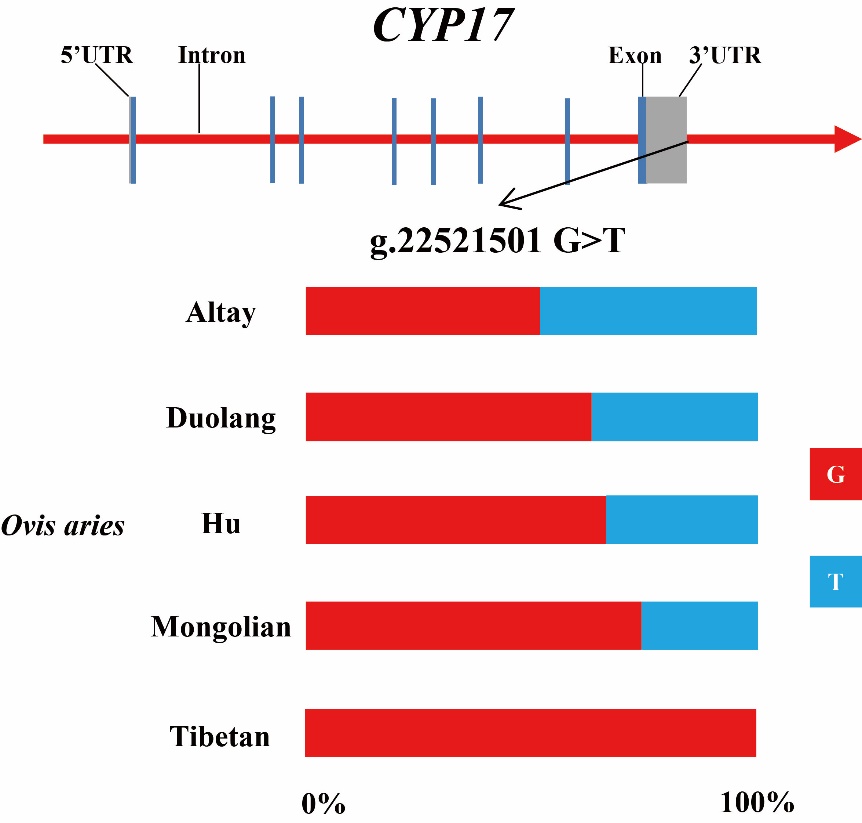


**Figure. S10** Structure and variation of the *CYP17* gene. The allele frequency of the g.22521501 G>T mutation in the downstream region of *CYP17* across five native Chinese sheep breeds.


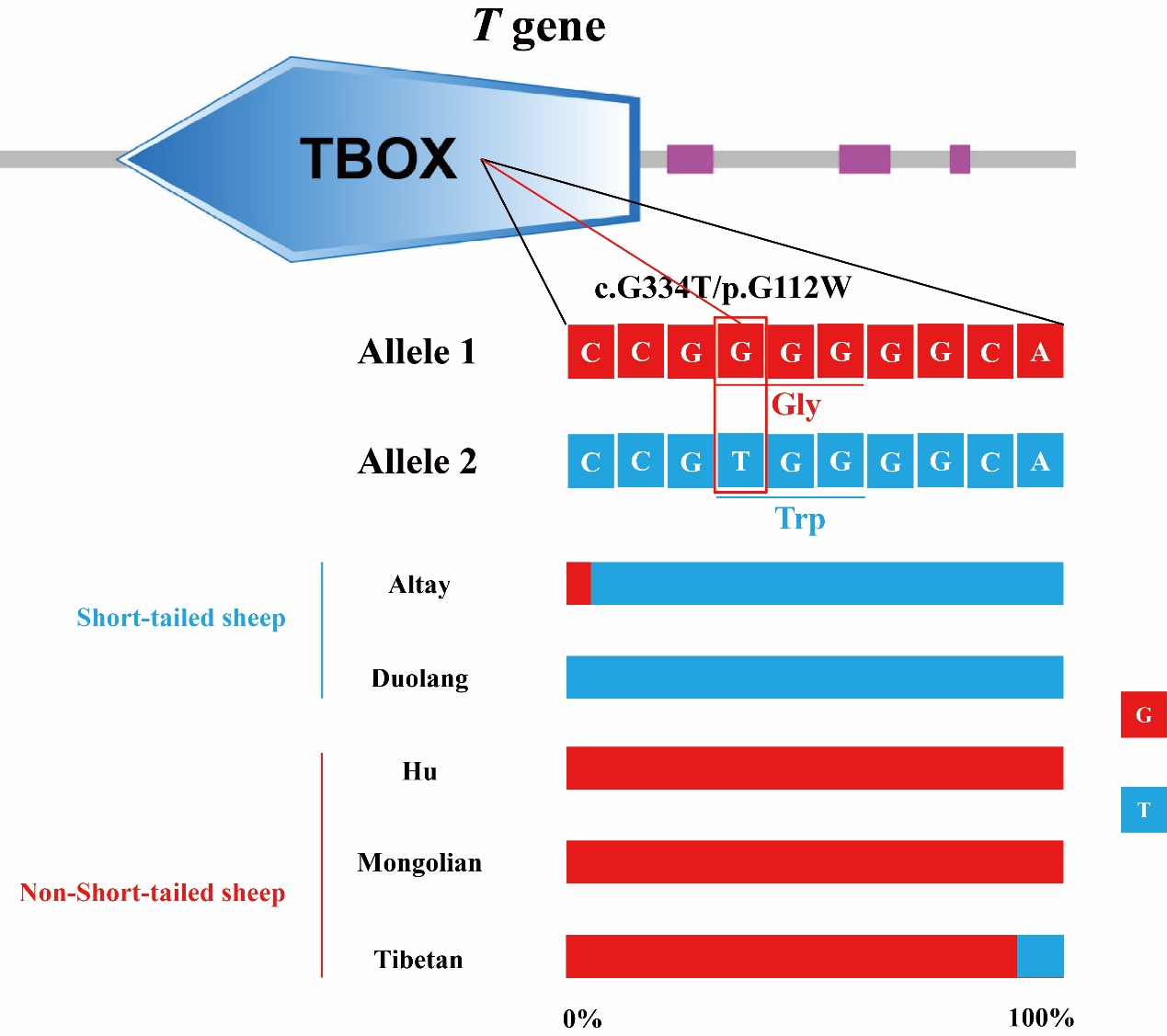


**Figure. S11** Structure and variation of the *T* gene. The blue pentagon represents the TBOX domain. The allele frequency of the c.G334T/p.G112W mutation of *T* across short-tailed and non-short-tailed sheep.
